# Fourth European Conference on Infections in Leukaemia (ECIL-4): Guidelines for Diagnosis and Treatment of Human Respiratory Syncytial Virus, Parainfluenza Virus, Metapneumovirus, Rhinovirus, and Coronavirus

**DOI:** 10.1093/cid/cis844

**Published:** 2012-09-28

**Authors:** Hans H. Hirsch, Rodrigo Martino, Katherine N. Ward, Michael Boeckh, Hermann Einsele, Per Ljungman

**Affiliations:** 1 Division of Infectious Diseases and Hospital Epidemiology, University Hospital Basel; 2 Transplantation and Clinical Virology, Department of Biomedicine, University of Basel, Switzerland; 3 Department of Hematology, Autonomous University of Barcelona, Hospital de la Santa Creu i Sant Pau, Barcelona, Spain; 4 Division of Infection and Immunity, University College London, United Kingdom; 5 Vaccine and Infectious Disease Division, Fred Hutchinson Cancer Research Center, Seattle, Washington; 6 Department of Internal Medicine II, Julius-Maximilians-University Würzburg, Germany; 7 Department of Hematology, Karolinska University Hospital; 8 Division of Hematology, Department of Medicine Huddinge, Karolinska Institutet, Stockholm, Sweden

**Keywords:** respiratory virus, transplantation, leukemia, bone marrow transplantation, hematopoietic

## Abstract

Community-acquired respiratory virus (CARV) infections have been recognized as a significant cause of morbidity and mortality in patients with leukemia and those undergoing hematopoietic stem cell transplantation (HSCT). Progression to lower respiratory tract infection with clinical and radiological signs of pneumonia and respiratory failure appears to depend on the intrinsic virulence of the specific CARV as well as factors specific to the patient, the underlying disease, and its treatment. To better define the current state of knowledge of CARVs in leukemia and HSCT patients, and to improve CARV diagnosis and management, a working group of the Fourth European Conference on Infections in Leukaemia (ECIL-4) 2011 reviewed the literature on CARVs, graded the available quality of evidence, and made recommendations according to the Infectious Diseases Society of America grading system. Owing to differences in screening, clinical presentation, and therapy for influenza and adenovirus, ECIL-4 recommendations are summarized for CARVs other than influenza and adenovirus.

## Teaser

Respiratory viruses have been recognized as a significant cause of morbidity and mortality in patients with leukemia and those undergoing hematopoietic stem cell transplantation. The risk for lower respiratory tract infections and a fatal outcome appears to depend on the intrinsic virulence of the specific community-acquired respiratory virus as well as factors specific to the patient, the underlying disease, and its treatment.

Community-acquired respiratory virus (CARV) infections include a variety of RNA viruses such as human orthymyxo-, paramyxo-, picorna-, and coronaviruses, and DNA viruses such as adeno-, boca-, and polyomaviruses [1, 2]. CARVs are detectable in the general population throughout the year, but some CARVs show a pronounced seasonality in temperate climates that can exceed epidemic thresholds [1, 3]. CARV respiratory tract infections (RTIs) range from asymptomatic replication to significant disease that typically affects the very young and the very old populations, patients with chronic medical conditions, and those with inherited, acquired, or drug-induced immune dysfunction [1, 4]. In the past 2 decades, CARV RTIs have been recognized as a significant cause of morbidity and mortality in patients with leukemia and those undergoing hematopoietic stem cell transplantation (HSCT) [5–9]. These patients are at increased risk for progression to lower RTI (LRTI) with clinical and radiological signs of pneumonia, respiratory failure, and fatal outcome. The risk of LRTI and fatal outcome appears to reflect the intrinsic virulence of specific CARVs as well as factors specific to the patient, the underlying disease, and its treatment.

To better define the impact of CARVs in leukemia and HSCT patients, and to improve their diagnosis and management, a working group of the Fourth European Conference on Infections in Leukaemia (ECIL-4) 2011 reviewed the literature on CARVs, graded the available evidence, and made recommendations according to the Infectious Diseases Society of America grading system (Supplementary Table 1). Because several aspects regarding influenzavirus and adenovirus differ substantially, including availability of vaccines and use of specific antivirals, and screening of high-risk patients for occurrence of gastrointestinal and disseminated disease, respectively, ECIL recommendations are summarized for CARVs other than influenza and adenovirus [10].

## METHODS

PubMed was searched using each of the following terms: respiratory virus, respiratory syncytial virus, metapneumovirus, parainfluenza, rhinovirus, enterovirus, picornavirus, coronavirus, polyomavirus, bocavirus; together with leukemia, or hematopoietic transplantation, or HSCT, or bone marrow transplantation, or cord blood. Published studies were identified and reviewed in August 2011. In June 2012, 5 additional papers and one paper in press were identified. The majority of publications are retrospective observational studies, while few prospective studies have been published dealing with this topic.

### CARV Diagnostic Considerations

The diagnosis of CARV RTI is dependent on the specimen and the laboratory assay(s) available. Potential specimens for diagnostic testing include nasopharyngeal aspirates, nasopharyngeal wash, swabs (preferably flocked for nasal sampling), tracheal aspirates, and bronchoalveolar lavage (BAL) [1, 11]. Pooling bilateral nasopharyngeal with throat swabs is often preferred over nasopharyngeal aspirates or nasopharyngeal wash for upper RTI (URTI), and BAL is preferred over tracheal aspirates for the diagnosis of LRTI. Laboratory tests include:

- Nucleic acid amplification testing (NAT), used as a generic term to describe molecular genetic tests such as polymerase chain reaction and others for the detection of viral DNA or RNA.
- Direct antigen detection (DAD), used as a generic term to describe direct detection of antigens in a specimen using specific antibodies in different assay formats (direct fluorescent antigen, enzyme-linked immunoassay, immune chromatography, etc).
- Virus isolation by cell culture (VIC), used as a generic term to describe cell culture for the isolation of infectious, replicating viruses. VIC is performed using conventional and/or shell vial cell culture techniques that can be combined with DAD for agent identification.

VIC has a higher clinical specificity for disease but requires a dedicated virology laboratory, is less sensitive than NAT, and has a comparatively long turn-around time of 2–5 days. DAD has a good clinical specificity, and a short turn-around time of <4 hours, but has a lower sensitivity compared with VIC and NAT [12–20]. NAT is often preferred because of a higher sensitivity, an acceptable turn-around time of <24 hours, in addition to the potential of quantifying viral loads, multiplexing with other infectious agents, detecting genetic variants, and molecularly characterizing nosocomial outbreaks in specialized laboratories.

### Definitions of CARV Infection and Disease

The detection of CARV in asymptomatic patients is increased when using sensitive NAT [16, 21, 22]. As outlined elsewhere previously [23], it is therefore important to distinguish between patients with CARV infection and CARV infectious disease. To provide a case definition comparable to one proposed by the European Centre for Disease Prevention and Control for influenza virus, the working group agreed to its adaptation for other CARVs in leukemia and HSCT patients (Table 1).

- URTI was defined as the detection of CARVs above and including the larynx (eg, in samples from nose, pharynx, larynx, conjunctivae, or sinuses).
- URTI disease (URTID) was defined as the detection of CARVs in upper respiratory tract fluid specimens together with symptoms and/or signs and other causes excluded.
- LRTI was defined as the detection of CARVs below the larynx (eg, in samples from trachea, bronchus, bronchoalveolar sites).
- LRTI disease (LRTID) was defined as pathological sputum production, hypoxia, or pulmonary infiltrates together with identification of CARVs in respiratory secretions, preferentially in samples taken from the sites of involvement (Table 1).

**Table 1.** Definitions of Community-Acquired Respiratory Virus Respiratory Tract Infectious Disease

Case Classification
Possible case: meeting the *clinical* criteria of RTID Probable case: meeting the *clinical* criteria of RTID together with an *epidemiological* link Confirmed case: meeting the *clinical* criteria of RTID and the *laboratory* criteria
Clinical criteria
New onset of symptoms AND at least 1 of the following 4 respiratory symptoms: ○ Cough ○ Sore throat ○ Shortness of breath ○ Coryza AND the clinician's judgment that the illness is due to an infection
Epidemiological criteria
An epidemiological link to human-to-human transmission (activity in the community, contact with visitor, another patient, or healthcare worker)
Laboratory criteria
Detection of CARV in a clinical specimen, preferably from the site of clinical involvement, by at least 1 of the following: ○ Virus isolation by cell culture ○ Direct virus antigen detection ○ Nucleic acid amplification testing AND exclusion of a major role of other etiologies

Abbreviations: CARV, community-acquired respiratory virus; RTID, respiratory tract infectious disease.

### Characteristics of Specific CARVs

#### (Human) Respiratory Syncytial Virus

Repiratory syncytial virus (RSV) falls into 2 distinct antigenic subgroups, A and B. Infections occur year-round but peak during the cold season, with increases in URTID (eg, sinusitis, rhinitis, and laryngitis) in young children, and LRTID (eg, bronchiolitis and pneumonia) in neonates [3, 24]. RSV RTI of patients with HSCT and/or other hematological diseases follow the community activity and reflect an increased risk of community-acquired, household, and nosocomial transmission [15, 25–27]. In the past, the standard diagnostic assays have been DAD and VIC, but these techniques have been replaced or complemented by NAT in many centers [14, 28]. RSV infections occur in 0.3%–2.2% of pediatric patients with acute myeloid leukemia [29] and in 1%–12% of adult patients with hematological malignancy and HSCT [8, 30–36]. Infection in the first 100 days after myeloablative allogeneic HSCT has been associated with an increased risk of persistent air flow decline at 1 year after transplant [37]. Progression to LRTID is observed in 38% (mean; range, 0%–68%) of leukemia and HSCT patients, with an average mortality of 32% (range, 0%–70%), as reviewed elsewhere [14, 38]. Risk factors for LRTID include infection during preengraftment, lymphopenia, older age, allogeneic HSCT, and severe immunodeficiency due to a range of contributing factors (Table 2). Although the risk of a poor clinical outcome progressively increases with overall falling absolute lymphocyte counts [9], varying thresholds of lymphopenia have been reported in clinical studies (0.3 to 0.1 × 10^9^/L) [8, 31, 32, 39]. Rapid diagnostics, infection control measures, and deferral of chemotherapy and/or HSCT are important considerations [22]. Corticosteroid treatment is a risk factor in leukemia patients [30], but the role of corticosteroids is controversial, since improving respiratory function has been seen despite increasing RSV loads and prolonged shedding [40, 41]. Currently, there is only limited evidence for effective treatments because of the lack of potent antiviral drugs and sufficiently powered, randomized controlled clinical trials (RCTs) [42, 43]. However, pooling of published studies suggests that treating URTID in HSCT and leukemia at risk for LRTID and treating manifest LRTID with ribavirin and intravenous immunoglobulin (IVIG) improves outcome [14, 33, 38]. It should be recognized that proper meta-analyses were not possible, and the results should therefore be interpreted with caution.

**Table 2.** Risk Factors of Respiratory Syncytial Virus–Associated Complications in Hematopoietic Stem Cell Transplantation Patients

Progression to LRTID
Lymphopenia <0.2 × 10^9^/L Older age Mismatched/unrelated donor Allogeneic HSCT <1 mo Neutropenia <500/µL No therapy with aerosolized ribavirin + IVIG
Mortality
Preengraftment Lymphopenia <0.2 × 10^9^/L Allogeneic HSCT <1 mo Severe immunodeficiency Older age (>65 y)

Abbreviations: HSCT, hematopoietic stem cell transplantation; IVIG, intravenous immunoglobulin; LRTID, lower respiratory tract infectious disease.

#### Human Parainfluenza Virus

The human parainfluenza virus (HPIV) species -1, -2, -3, and -4 cause mild URTID throughout the year, but type-specific seasonal increases of URTID and LRTID with laryngotracheitis, bronchiolitis, and pneumonia are seen in 15% of infected children during autumn and spring [3, 44]. Diagnosis of HPIV infection has been largely made using DAD or VIC covering HPIV-1, -2, and -3, but is increasingly replaced by NAT also identifying HPIV-4. In adult and pediatric leukemia and HSCT patients, symptomatic HPIV infections have been reported to range from 2% to 7%, of which at least one-third are manifest as LRTID [9, 32, 45–49]. Among the pediatric patients, 90% of HPIV infections were deemed to be community-acquired. Given an estimated incubation period of 2.6 days (95% confidence interval [CI], 2.1–3.1) [50] and a high rate of 17.9% asymptomatic shedding [36], outpatient and nosocomial outbreaks are not infrequent, indicating the need for infection control strategies [18, 46–48, 51–56]. In HSCT recipients with URTID and LRTID, HPIV-3 is the most commonly detected type in children as well as in leukemia and HSCT patients (80%–90%) followed by HPIV-1 and -2 [44, 45, 57, 58]. Nonmyeloablative conditioning has been associated with HPIV URTI after 30 days after transplant [48]. URTI has been associated with significant airflow decline in 40% of patients [37], which may progress to LRTID in 13%–37% and a fatal outcome in 10%–30% [45, 57]. Reported risk factors for LRTID are higher corticosteroid exposure, neutropenia, lymphopenia, infection early after allogeneic HSCT, a higher APACHE II score, and coinfections [32, 45, 47, 48, 57–60]. Treatment options are limited by the lack of effective agents and RCTs, although some centers consider treating HPIV URTID in patients with risk factors for LRTID and HPIV LRTID, with ribavirin and/or IVIG [8, 18, 31, 36, 37, 48, 61–64]. Bronchiolitis obliterans syndrome and obstructive airflow decline have been associated with HPIV infection within the first 3 months after allogeneic HSCT, which persisted at 1 year after transplant [37, 65].

#### Human Metapneumovirus

Human metapneumovirus (HMPV) is a paramyxovirus closely related to RSV, causing increases in URTID and tracheobronchitis in 5%–20% of children and adults during winter. HMPV infection is commonly diagnosed by NAT, and rates range from 2.5% to 9% during the first 2 years after allogeneic HSCT [32, 66–68]. Asymptomatic and prolonged shedding has been reported in HSCT patients [36, 69, 70]. HPMV URTID in HSCT patients can present with flu-like symptoms [66, 67]. In HSCT patients with pneumonia, HMPV is frequently codetected with other pathogens, including bacteria, fungi, and other CARVs, as well as cytomegalovirus, all which obscure the attributable morbidity [32, 66]. Recipient cytomegalovirus seropositivity was a risk factor in one study of HSCT patients [36]. Single cases of severe disease and fatal outcome have been reported [71, 72]. No general recommendation for treatment can currently be made, although some centers consider treating HMPV LRTID with ribavirin and/or IVIG despite the lack of supporting studies [19, 26, 32, 66, 67].

#### Human Coronavirus

Human coronaviruses (HCoVs) circulate throughout the year with a slight predominance in winter, presumably causing 10%–30% of cases of the “common cold.” HCoVs are divided into group 1–like (CoV-229E and -NL63) and group 2–like (CoV-OC43 and -HKU1) agents that are molecularly distinct. Although VIC and DAD are available, most centers use NAT in multiplex formats, reporting rates of 5.7% among acutely symptomatic patients. The incubation period has been estimated as 3.2 days (95% CI, 2.8–3.7) [50] followed by a median detectability of 2–3 weeks. URTID with rhinitis, pharyngitis, and laryngitis is the most common manifestation. Cases of LRTID with bronchitis, bronchiolitis, and pneumonia have been reported in very young (age <1 year) and/or in immunodeficient patients [73–75]. In HSCT patients, HCoV has been detected in 6.7%–15.4%, but asymptomatic shedding may be as high as 41% [21]. In symptomatic HSCT patients, coinfections with other pathogens are frequent. LRTID and pneumonia with fatal outcome occur rarely [76]. General recommendations for treatment are limited in view of the largely benign course, the lack of effective antiviral agents, and appropriate clinical studies [21, 75, 77, 78].

#### Human Rhinovirus

Human rhinoviruses (HRhVs) belong to the Picornaviridae family and are divided into 3 species called A, B, and C encompassing >100 serotypes. HRhVs circulate throughout the year and are the most common cause of URTID (rhinorrhea, postnasal drip, cough) and occasionally (tracheo-)bronchitis [1]. The incubation period has been estimated as 1.9 days (95% CI, 1.4–2.4) [50]. Diagnosis largely depends on NAT, although DAD for rapid testing and VIC are performed in specialized laboratories. In allogeneic HSCT recipients, HRhVs have been identified as the most frequent CARVs, reaching a cumulative incidence as high as 22.3% by day 100 [21], with detection rates of up to 40% among symptomatic HSCT patients [34]. HRhV infection may be asymptomatic in 13% of HSCT patients, and prolonged shedding over 4 weeks is frequent, with coinfections with other CARVs occurring in 19% of patients [79]. One study reports that higher HRhV loads correlate with symptomatic presentations [80]. LRTID with frank pneumonia is rare and may occur in <10% of allogeneic HSCT infected with HRhV, usually in myeloablative conditioning, with an estimated mortality of <10% [32, 59, 79, 81]. The role of HRhV treatment is limited by the lack of agents and clinical trials.

#### Other CARVs

Human enteroviruses (HEnV), encompassing at least 66 serotypes, also belong to the Picornaviridae family. HEnVs are detected in <5% of hematological patients with URTID, which may progress to LRTID in 13% [9, 34, 79]. Although some HEnVs are identifiable by VIC, current laboratory diagnosis relies mostly on NAT, which may also be designed to detect other picornaviruses such as HRhV or parechoviruses. Lymphopenia of <500/µL is a risk factor for LRTID in HSCT patients.

Human bocavirus (HBoV) and human polyomavirus (HPyV) infections have been detected in patients with hematological malignancies or HSCT [82]. However, studies of cases with a well-documented clinical course and proven disease by histopathology are missing. Accordingly, risk factors for disease and the need for therapy are not well defined. HBoV belongs to the Parvoviridae family and is detected in 5% of children with RTI. HBoV has been frequently codetected with other viral agents, preventing an unequivocal attribution to URTID or LRTID. In BAL from adult patients, HBoV was detected in 0%–3% of cases. Recent studies suggest that HBoV loads >5 log_10_ copies/mL in respiratory fluids are more likely to indicate clinically significant replication [83]. Disseminated HBoV infection has been reported, but the clinical interpretation of NAT signals, even when found in blood or organ sites, may be difficult, since its prolonged persistence has been described akin to parvovirus B19 [84].

HPyV RTIs include KIPyV and WUPyV, which have been detected in 0.2% and 1.4% of children with acute URTID, respectively [85]. KIPyV has been detected more frequently in respiratory fluids of HSCT patients (17%) compared with other patients (5%) [86]. In symptomatic children with leukemia or HSCT, higher viral loads in BAL have been reported [87]. In a large prospective study of 222 HSCT patients, KIPyV and WUPyV showed a cumulative incidence of 26% and 8%, respectively, after 1 year, with no seasonal pattern, but an increased rate in patients <20 years of age (hazard ratio, 4.4 and 4.6, respectively) [88]. Sputum production and wheezing were associated with KIPyV or WUPyV detection, but not with graft-vs-host disease, cytomegalovirus reactivation, neutropenia, lymphopenia, hospitalization, or death [88]. Pending further studies, routine testing for KIPyV and WUPyV cannot be recommended, and there are currently no data supporting the treatment of KIPyV or WUPyV LRTID [86].

### ECIL-4 Recommendations on Prevention of CARV Infection

The working group recognizes that the person-to-person transmission of CARVs should lead to measures for their prevention through infection control measures (Table 3). These recommendations should be implemented at the level of patients, relatives, and healthcare workers, both inside and outside of medical institutions (Table 3).

**Table 3.** Recommendations on Prevention of Community-Acquired Respiratory Virus Infection

• It is recommended that patients and contact persons should adhere to good personal hygiene, including frequent hand washing, covering the mouth when coughing and sneezing, and disposing safely of oral and nasal secretions (***AII***).
• Leukemia patients and HSCT patients should avoid contact with individuals with RTI in the hospital and in the community (***AII***).
• Young children should be restricted from visiting patients and wards because of the higher risk of CARV exposure, prolonged shedding, and ease of transmission (***BII***).
• All visitors and HCWs with RTI should be restricted from access to patients and wards (***AII***).
• Inside care facilities, infection control measures should be applied to leukemia and HSCT patients with RTI, including isolation rooms and application of strict protection measures (gloves, gowning, masks, eye protection) for HCWs and visitors (***AII***).
• Outpatients with RTI should be seen and treated in accordance with infection control measures, ie, in facilities and rooms separated from other HSCT and leukemia patients (***AII***).

See Supplementary Table 1 for the Infectious Diseases Society of America grading system.

Abbreviations: CARV, community-acquired respiratory virus; HCW, healthcare worker; HSCT, hematopoietic stem cell transplantation; RTI, respiratory tract infection.

Administration of IVIG preparations to HSCT and leukemia patients with hypogammaglobulinemia <4 g/L may reduce the risk of morbidity or mortality secondary to CARV RTIDs (***CIII***). During RSV outbreaks in the community indicating an increased risk of exposure, the use of intravenous monoclonal antibody specific for the RSV-F protein (palivizumab) may be considered for pediatric patients aged <2 years as monthly prophylaxis (***CIII***), but it is not indicated in other patient groups.

### ECIL-4 Recommendations for Diagnosis of CARV Infection

To balance costs and clinical benefit, screening all patients for CARVs is currently not indicated unless indicated in the context of an infection control investigation of nosocomial transmission and prevention, and thus laboratory testing should focus on symptomatic patients (Table 4). Taking into account the clinical impact of CARVs in HSCT and leukemia patients and the differences among centers in the technical and financial resources for comprehensive CARV diagnostics by multiplex NAT, the working group recommends prioritizing laboratory tests for specific CARVs such as influenza, RSV, and HPIV (Table 4).

**Table 4.** Recommendations for Diagnosis of Community-Acquired Respiratory Virus Infection

• HSCT candidates or HSCT recipients with URTID or LRTID should be tested for CARVs to guide infection control measures, treatment, and decisions regarding deferral of chemotherapy or HSCT (***AII***).
• Specimens should preferably be taken from the site of clinical involvement, preferably pooled swabs for URTID, or BAL for LRTID, (or tracheal aspirate if BAL is not available) (***BII***).
• First-line diagnostic testing should be performed for influenza A and B, RSV, and HPIV (***AII***).
• Testing for other CARVs^a^ should be considered according to risk of exposure and the local epidemiology, or if testing for the first-line CARVs is negative (***BIII***).
• Patients with LRTID should be considered for BAL and broader diagnostic testing including lung biopsy as clinically indicated (***BII***).

See Supplementary Table 1 for the Infectious Diseases Society of America grading system.

Abbreviations: BAL, bronchoalveolar lavage; CARV, community-acquired respiratory virus; HPIV, human parainfluenza virus; HSCT, hematopoietic stem cell transplantation; LRTID, lower respiratory tract infectious disease; RSV, respiratory syncytial virus; URTID, upper respiratory tract infectious disease.

^a^ Including human enterovirus, human metapneumovirus, human rhinovirus, human coronavirus, and human adenovirus.

### ECIL-4 Treatment Recommendations for CARV Infection

Reflecting the clinical impact compared to other CARVs, the working group distinguishes the need of treatment for influenza A and B [10], RSV and HPIV, taking into account the higher risk for poor outcome in specific patient groups. The treatment of RSV and HPIV may involve the deferral of conditioning therapy, treatment with aerosolized ribavirin, or off-label use of systemic ribavirin, whereas no general recommendations for other CARVs can be made at this time (Table 5).

**Table 5.** Recommendations for Community-Acquired Respiratory Virus Treatment in Hematopoietic Stem Cell Transplantation and Leukemia Patients

• Deferral of conditioning therapy should be considered for patients with CARV RTID planned for allogeneic HSCT (***BII***)
• Deferral of conditioning/chemotherapy could be considered for patients with CARV RTID scheduled for chemotherapy of hemato-oncological diseases (***BIII***)
• Patients with RSV URTID undergoing allogeneic HSCT or recipients of allogeneic HSCT with risk factors for progression to RSV LRTID and death should be treated with aerosolized or systemic ribavirin and IVIG (***BII***)
• For allogeneic HSCT patients with HPIV LRTID, treatment with aerosolized or systemic ribavirin and IVIG may be considered (***BIII***)
• For allogeneic HSCT patients with CARV URTID or CARV LRTID other than RSV or HPIV, aerosolized or systemic ribavirin and IVIG treatment cannot be recommended (***CIII***)

See Supplementary Table 1 for the Infectious Diseases Society of America grading system.

Abbreviations: CARV, community-acquired respiratory virus; HPIV, human parainfluenza virus; HSCT, hematopoietic stem cell transplantation; IVIG, intravenous immunoglobulin; LRTID, lower respiratory tract infectious disease; RSV, respiratory syncytial virus; RTID, respiratory tract infectious disease; URTID, upper respiratory tract infectious disease.

The corresponding modalities of RSV therapy and systemic ribavirin are summarized in Tables 6 and 7, respectively. The working group is cautious about the use of intravenous monoclonal antibody specific for the RSV-F protein, because existing data outside of single case reports do not support its beneficial effect and the cost is very high. Therefore, only very young (age <2 years) allogeneic HSCT patients with LRTID or at high risk for progression to RSV LRTID might be considered for treatment with intravenous monoclonal antibody specific for the RSV-F protein (eg, palivizumab 15 mg/kg body weight) (***CIII***; Supplementary Table 1), while this drug should not be considered in other patient groups.

**Table 6.** Recommendations for Respiratory Syncytial Virus Treatment in Hematological Patients

• For treatment of RSV, aerosolized ribavirin can be administered as 2 g for 2 h every 8 h or as 6 g over 18 h/d for 7–10 d (***BII***).
• For treatments using aerosolized ribavirin, appropriate precautions should be applied to avoid environmental exposure and thereby potentially teratogenic effects in pregnant healthcare workers and visitors (***AII***).
• Patients on aerosolized ribavirin should be monitored and treated for adverse events including claustrophobia, bronchospasm, nausea, conjunctivitis, and declining pulmonary function (***BII***).
• For treatment of RSV, systemic ribavirin can be administered orally (***BIII***) or intravenously for patients unable to take oral medication (10–30 mg/kg body weight in 3 divided doses) **(*CIII***).
• Patients on systemic ribavirin should be monitored and treated for adverse events including hemolysis, abnormal liver function tests, and declining renal function (***BIII***).
• For allogeneic HSCT patients with RSV LRTID or at high risk for RSV LRTID, aerosolized or systemic ribavirin therapy may be combined with IVIG or anti-RSV-enriched antibody preparations (***BIII***).

See Supplementary Table 1 for the Infectious Diseases Society of America grading system.

Abbreviations: HSCT, hematopoietic stem cell transplantation; IVIG, intravenous immunoglobulin; LRTID, lower respiratory tract infectious disease; RSV, respiratory syncytial virus.

**Table 7.** Use of Systemic Ribavirin for Respiratory Syncytial Virus or Human Parainfluenza Virus Respiratory Tract Infectious Diseases^a^

Oral or intravenous ribavirin maximal dosing 10 mg/kg body weight every 8 h for adults	
Day 1: Start with 600 mg loading dose, then 200 mg every 8 h	
Day 2: 400 mg every 8 h	
Day 3: Increase the dose to a maximum of 10 mg/kg body weight every 8 h	
In case of adverse events:	Decrease dose or discontinue ribavirin
Creatinine clearance:	Oral or intravenous administration
30–50 mL/min	Maximal 200 mg every 8 h
10–30 mL/ min	No recommendation can be given^b^

^a^ Modified after [14].

^b^ Some experts use 200 mg once daily under close clinical and laboratory monitoring.

Withholding treatment for RSV infection might be considered for selected stable leukemia and HSCT patients after careful evaluation of risk factors for morbidity and mortality and the possibility of appropriate follow-up visits considering, for example, remission of underlying disease, absence of immunosuppressive drug treatment, absence of the risk factors associated with LRTID, or mortality (***CIII***). Although some centers would treat patients with HPIV URTID and risk factors listed in Table 3, treatment of HPIV URTID is not generally recommended given the clinically undefined risk and benefit ratio (***CIII***).

Overall, the evidence is more limited for patients with autologous HSCT and/or hemato-oncological disease.

Infection control measures should be applied to patients undergoing autologous HSCT or chemotherapy for hemato-oncological diseases with CARV URTID or LRTID (***BIII***).

Deferral of conditioning/chemotherapy should be considered for patients with CARV-RTID scheduled for autologous HSCT or chemotherapy for hemato-oncological diseases (***BIII***). Treatment of CARV RTID other than influenza is not generally recommended for patients undergoing autologous HSCT or chemotherapy for hemato-oncological diseases (***CIII***).

## DISCUSSION AND OUTLOOK

The working group acknowledges that despite the growing awareness of infections by CARVs in HSCT and leukemia patients, well-designed studies are largely lacking that evaluate diagnostic and therapeutic strategies for CARV. On the diagnostic level, studies are needed to identify the most appropriate diagnostic test and specimen from the upper and lower respiratory tracts. The detection of CARVs in peripheral blood has been associated with significant LRTID, disseminated disease, and poor outcome, but requires evaluation by specifically designed studies. There is interest to identify and confirm risk factors of severe disease and poor outcome and to evaluate laboratory markers of virus-specific immunity as surrogate markers of disease and recovery. The recent attempts to use RSV loads as a virological surrogate marker of antiviral treatment by small interfering RNA and/or clinical outcome may have a pacemaker role for other CARVs [89]. Importantly, the currently available treatments for CARV URTID and LRTID lack rigorous evaluation in appropriately sized, prospective randomized controlled trials. This is needed for comparing aerosolized ribavirin with systemic (oral) ribavirin; for evaluating the role of expensive IVIG preparations in combination with ribavirin; and for determining the use of intravenous monoclonal antibody specific for the RSV-F protein (palivizumab, motavizumab) as postexposure prophylaxis for high-risk patients as well as therapy for RSV URTID and LRTID. The development of vaccines is seen as an important area of research. Finally, a better understanding of the indirect alloimmune pathology of CARVs on clinical outcome is important [65], but also depends on a better definition of the direct viral impact.

## Supplementary Material

Supplementary Data
