# Supplementary material for: Fourth European Conference on Infections in Leukaemia (ECIL-4): Guidelines for Diagnosis and Treatment of Human Respiratory Syncytial Virus, Parainfluenza Virus, Metapneumovirus, Rhinovirus, and Coronavirus
Source: Clin Infect Dis. 2012 Sep 28;56(2):258–66. doi: 10.1093/cid/cis844 (PMC3526251; doi:10.1093/cid/cis844)
Supplement: Supplementary Data [file cis844_Supplementary_Data.zip › cis844supp.doc]

**Supplement**

**Table 1: Infectious Disease Society of America - United States Public Health Service grading system for ranking recommendations**

| **Quality of evidence** | **Strength of recommendation** |
| --- | --- |
| I  Evidence from ≥ 1 properly randomized, controlled trial | A  Good evidence to support a recommendation for use |
| II  Evidence from ≥ 1 well-designed clinical trial, without randomization; from cohort or case-controlled analytic studies (preferably from >1 center); from multiple time-series; or from dramatic results from uncontrolled experiments | B  Moderate evidence to support a recommendation for use |
| III  Evidence from opinions of respected  authorities, based on clinical experience, descriptive studies, or reports of expert committees | C  Poor evidence to support a recommendation |
