# Supplementary material for: Fourth European Conference on Infections in Leukaemia (ECIL-4): Guidelines for Diagnosis and Treatment of Human Respiratory Syncytial Virus, Parainfluenza Virus, Metapneumovirus, Rhinovirus, and Coronavirus
Source: Clin Infect Dis. 2012 Sep 28;56(2):258–66. doi: 10.1093/cid/cis844 (PMC3526251; doi:10.1093/cid/cis844)
Supplement: Supplementary Data [file cis844_Supplementary_Data.zip › cis844supp1.doc]

**Acknowledgments**

The ECIL is a joint venture of the Infectious Diseases Working Party of the European Blood and Marrow Transplantation Group, of the Infectious Diseases Group of the European Organization for Research and Treatment of Cancer, of the Immunocompromised Host Society, and of the European Leukemia Net.

We are indebted to the participants of the ECIL 4 meeting:

Hamdi Akan, Murat Akova, Turkey; Dina Averbuch, Israel; Rose-Mary Barnes, United Kingdom; Nicole Blijlevens, The Netherlands; Thierry Calandra, Switzerland; Elio Castagnola, Simone Cesaro, Italy; Catherine Cordonnier, France; Oliver Cornely, Germany; Jean-Hughes Dalle, France; Rafael de la Camara, Spain; Emma Dellow (Gilead Sciences) United Kingdom; Peter Donnelly, The Netherlands; Lubos Dgrona, Slovakia; Hermann Einsele, Germany; Dan Engelhard, Israel; Bertrand Gachot, France; Corrado Girmenia, Italy; Andreas Groll, Germany; Ingeborg Gyssens, The Netherlands; Werner Heinz, Germany; Raoul Herbrecht, France; Hans H. Hirsch, Switzerland; William Hope, United Kingdom; Petr Hubacek, Czech Republic; Chris Kibbler, United Kingdom; Galina Klyasova, Russia; Sean Knox (Astellas Pharma), United Kingdom; Michal Kouba, Czech Republic; Catherine Lagrou, Belgium; Thomas Lernbecher, Germany; Per Ljungman, Sweden; Johan Maertens, Belgium; Oscar Marchetti, Switzerland; Rodrigo Martino, Spain; Georg Maschmeyer, Germany; Tamas Masszi, Hungary; Suzanne Matthes-Martin, Austria; Malgorzata Mikulska, Alessandra Micozzi, Italy; Bilal Mohty, Switzerland; Patricia Munoz, Spain; David Nadal, Christina Orasch, Switzerland; Zdenek Racil, Czech Republic; Patricia Ribaud, France; Valérie Rizzi-Puechal (Pfizer), France; Emmanouil Roilidis, Greece; Janos Sinko, Hungary; Jan Styzynscki, Poland; Alina Tanase, Hungary; Mario Tumbarello, Italy; Paul Verweij, The Netherlands; Claudio Viscoli, Italy; Kate-Nora Ward, United Kingdom; Aafje Warris, The Netherlands; Craig Wood (Merck), USA.

The ECIL Organization Committee is indebted to the companies who supported the ECIL 4 meeting in September 9-10, 2011, through educational grants: Astellas Pharma, Gilead Sciences, Merck, Novartis, and Pfizer.

We are also thankful to Jean-Michel Gosset and the staff of KOBE, group GL Events, Lyon, France, for the organization of the meeting.
